# Fabrication and characterization of impact-resistant core-spun yarn fabrics with a hydroxylated fullerene-strengthened shear thickening fluid†

**DOI:** 10.1039/d2ra01095j

**Published:** 2022-04-25

**Authors:** Wenhua Cai, Rong Zhang, Xuechen Wang, Xingxiang Zhang

**Affiliations:** School of Material Science and Engineering, Tiangong University Tianjin 300387 China caicaiwenhua@163.com; Hangyu Lifesaving Equipment Co., Ltd Xiangyang 441002 China zhang_rong999@163.com; National & Local Joint Engineering Research Centre of Advanced Fibre and Textile Composite Technology, Tiangong University Tianjin 300387 China zhangtjpu@hotmail.com +86-022-83955054

## Abstract

Shear thickening fluid (STF) is investigated to strength soft armor; however, its impact resistance still does not meet practical needs. In this work, a small amount of hydroxylated fullerene (C_60_) was mixed with STF to improve the thickening ratio. First, furfuryl alcohol (FA) was grafted onto C_60_ through a Diels–Alder (D–A) reaction to improve the dispersity of C_60_ in the STF. Sheath-core composite fibers (polyketone (PK) as the sheath and STF as the core) were then fabricated by coaxial electrospinning. Finally, composite fibers containing STF and C_60_ were wrapped on the surface of aramid yarns to fabricate a core-spun yarn. Under impact, these core-spun yarns manifested the characteristics of aramid fibers and the thickening advantages of the STF, solving problems of the hygroscopicity, migration, and leakage of STF. In addition, the content of STF was also greatly increased. The spike punching resistance of the core-spun yarn fabric is about 2.8 times that of the aramid fabric (AF) under the same area density. Impact-resistant core-spun yarn fabrics could provide a new direction for the development of soft armor.

## Abstract 2

Shear thickening fluid (STF) is investigated to strength soft armor; however, its impact resistance still does not meet practical needs.

## Graphical abstract

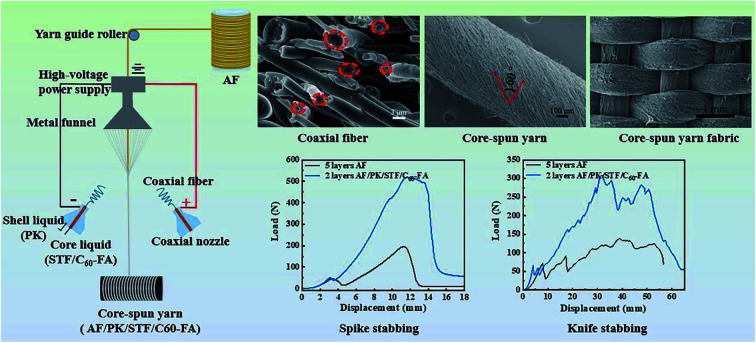

## 1. Introduction

Body armor is designed to protect soldiers from bullets and other weapons. Soft body armor is particularly important personal protective equipment and plays an important role in protecting the lives of soldiers and police and reducing the degree of casualties. Aramid fibres are extensively used to make bulletproof military equipment because of their high strength, high modulus, light weight, and good toughness.^1–3^

The viscosity of shear thickening fluid (STF) increases rapidly when an impact exceeds the critical shear thickening rate.^4–8^ In 2003, Wagner *et al.*^4^ impregnated STF on aramid fabric (AF) for the first time to improve the impact resistance. The influences of the rheological properties of STF,^9,10^ the impregnation method,^8^ and the lamination angle of fabrics^5,6^ on the impact resistance of composite materials have been extensively discussed since then. The addition of STF can significantly improve the impact resistance of fabrics, however, it manifests little effect on the thickness, weight, and stiffness of woven fabrics. Shear thickening^11^ and the friction improvement between yarns^12^ are the main STF mechanisms to enhance the impact resistance of woven fabrics. However, when STF is used for a long time, it absorbs moisture in the air and the shear thickening performance attenuates.^7,13^ In addition, STF will migrate or leak from the fabric surface. Wu *et al.*^14^ used STF as the core in a polyurethane grid and noticed that it had a positive effect on dynamic impact resistance. However, the specimen thickness was up to 2–3 cm. Warren *et al.*^15^ used STF as the core to design a spacecraft shielding component, and glued the panel on both sides with an adhesive, encapsulating the STF inside. STF played a significant role in the impact process. These ideas solve the current shortcomings of STF in the application, but for human body protection, this type of product loses its flexibility.

The order-disorder theory and the hydrocluster theory are mainly used to explain the shear thickening behavior of STF.^16^ The order-disorder theory^17,18^ describes the transition from an ordered layer to a disordered structure with the increase of shear rate, leading to an increase of intergranular resistance. The hydrocluster theory^19^ describes the tendency of particles to form clusters under shear, leading to an increment in resistance between particles. Hoffman^20^ proposed the theory of particle physical contacts. The contact force generated by direct friction plays an important role in continuous and discontinuous shear thickening, and the contact force is the main factor of shear thickening.^21,22^

Graphene nanoplatelets (GNs),^9,23,24^ carbon nanotubes (CNTs),^10,25–27^ or both^28–30^ are also added to STF to achieve an enhanced thickening performance. Sha *et al.*^28^ reported that CNTs contributed more significantly to the improvement of shear thickening behaviour than GNs at the same mass fraction. Wei *et al.*^27^ noticed that multi-walled carbon nanotubes (MWCNTs) could adsorb silica nanoparticles and form a new MWCNTs particle group. When the loading reached 8 wt%, STF had the best shear thickening effect. The addition of graphene oxide (GO) and CNTs increases the initial viscosity of STF and lower the thickening ratio due to the intertangling of CNTs and the large aspect ratio of GO.^31^ Fullerene (C_60_) has a special football shape with a molecular diameter of only 0.71 nm and a compressive strength of 2.5 GPa and plays an obvious role in increasing the contact force between STF particles.

In this work, a small amount of hydroxylated C_60_ strengthened STF was encapsulated in the fibre core by coaxial electrospinning. Composite fibres were wrapped on the surface of aramid yarns to fabricate core-spun yarns, which were then woven into a fabric. Under an impact, the friction between aramid yarns greatly increased, thereby improving the impact resistance of the fabric. Therefore, core-spun yarn could solve common problems of the hygroscopicity, migration, and leakage of STF impregnated on the surface of aramid fibres cleverly.

## 2. Experimental section

### 2.1 Materials

Nano silica (SiO_2_, particle size = 15 nm), hexafluoroisopropanol (HFIP), and furfuryl alcohol (FA) were procured from Shanghai Aladdin Biological Technology Co. Ltd, China. Polyethylene glycol 400 (PEG 400), dimethyl sulfoxide (DMSO), and absolute ethanol were purchased from Tianjin Kemiou Chemical Reagent Co. Ltd, China. Fullerene (C_60_, 99.5%) was obtained from Sigma-Aldrich. Polyketone (PK, M330) was purchased from Hyosung Corp., Korea. The polyacrylate coating agent TJ-26 was supplied by Shijiazhuang Federal Kete Chemical Co. Ltd, China. Aramid fabric TH5108 (area density = 210 ± 10 g m^−2^) and yarn were obtained from Yantai TAPARON Advanced Manufacturing Technology Co. Ltd, China.

### 2.2 Fabrication of hydroxylated C_60_

To improve the dispersibility of C_60_ in STF, FA was grafted on C_60_ through the Diels–Alder (D–A) reaction.^32,33^ The influence of solvent on the process of D–A reaction was discussed. The first group: 200 mg of C_60_ were added to 5.88 g of FA, and the resultant product was named C_60_-FA. 20 ml of DMSO (solvent) were added on the basis of the first group, and the resultant product was named C_60_-FA/DMSO. The reaction was conducted at 120 °C for 72 h. The final product was repeatedly centrifuged and washed with absolute ethanol and then dried in a vacuum oven at 80 °C until the mass was a constant.

### 2.3 Coaxial electrospinning of sheath/core composite fibres

First, SiO_2_ were dried under vacuum at 120 °C for 12 h and gradually added to PEG400 under mechanical stirring to obtain STF with 20 wt% of SiO_2_, which was left for 24 h to remove air bubbles. Furthermore, 0.1–0.4 wt% of C_60_-FA were added to STF under magnetic stirring for 4 h to obtain STF/C_60_-FA, which was left for 12 h and then tested for rheological properties. PK M330 pellets were dried in an oven at 60 °C for 10 h, then dissolved in 11 wt% of HFIP at 25 °C, and used as the sheath of sheath/core coaxial fibres. PK/STF coaxial composite fibres with STF as the core material and PK/STF/C_60_-FA coaxial composite fibres with 0.3 wt% C_60_-FA-enhanced STF as the core material were electrospun. According to previous experiments,^34^ a 5 ml medical syringe was used, and the feed rates of the core and sheath materials were 0.0007 mm min^−1^ and 0.0028 mm min^−1^, respectively. The positive and negative DC voltages were set to 13 kV, and the coaxial needle type was 17/23.

### 2.4 Fabrication of core-spun yarn fabric

**Fig. 1:**
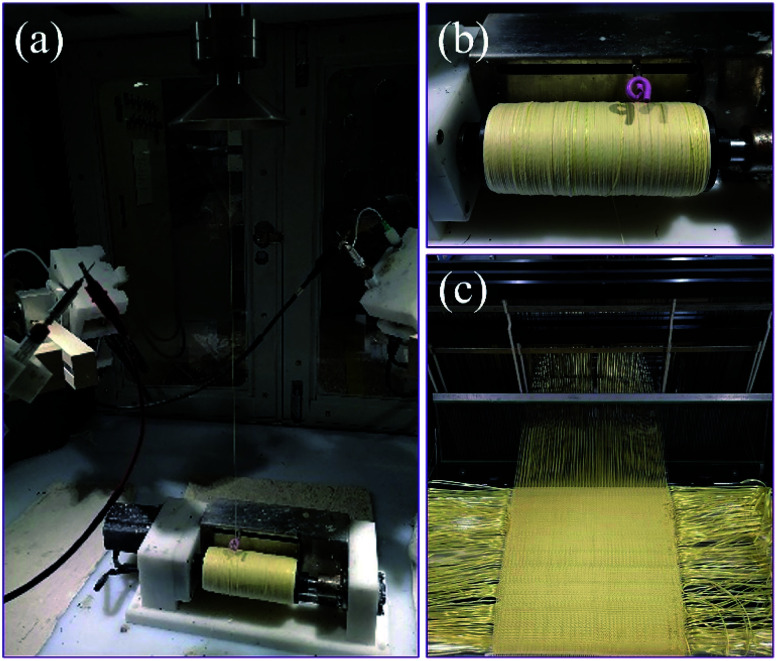
(a) Fabrication process of core-spun yarn, (b) collected core-spun yarns, and (c) core-spun yarn fabric weaving process.

Fig. 1(a) displays the fabrication process of core-spun yarns, and Fig. 1(b) presents the collected core-spun yarns. Under the condition of coaxial electrospinning, the flat plate collector was converted into a conical collector. The vertical distance between the collector and the needle was about 20 cm. Raw aramid yarns were passed through the middle of the collector and were connected to the yarn collecting roller. The elevation angle between the needle and the collector was adjusted to a stable spinning state between 45° and 60°. The speed of the yarn collector was set to 300 rpm, and the collection rate of core-spun yarn was 1.2 m min^−1^. The yarn linear density increased from 169 tex to 185 tex. To verify the role of STF in the impact experiment, two types of core-spun yarns were fabricated: (i) AF/PK with AF as the core and PK as the sheath and (ii) AF/PK/STF/C_60_-FA with AF as the core and PK/STF/C_60_-FA as the sheath. A video of the fabrication process of core-spun yarns is presented in ESI.†

The collected core-spun yarn was used to fabricate core-spun yarn fabric. Plain weave fabric exhibits better energy absorption performance than twill and satin weave fabrics because its higher interlacing points can transmit stress to a larger fabric area through more secondary yarns during energy dissipation.^35,36^ The TJ-26 coating agent was used to fix composite fibres on the core-spun yarn surface to avoid abrasion during the weaving process. The linear density of treated yarn was about 188 tex. Fig. 1(c) presents the yarn weaving process. The warp and weft densities were calculated by eqn (1).1
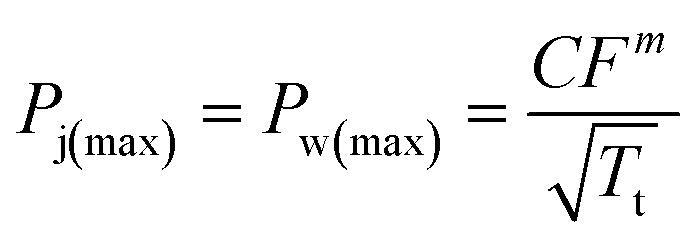
where, *P*_j(max)_ and *P*_w(max)_ are the maximum warp and weft density of the 10 cm fabric, respectively, *T*_t_ is the warp and weft yarn density, *F* is the average floating length of the fabric, *m* is the weave coefficient of the fabric, and *C* is the material coefficient of the fabric (*m* = 1 and *C* = 1300).

### 2.5 Material characterization

The surface and cross-sectional morphologies of the as-prepared fibres were characterized by a field-emission scanning electron microscope (FE-SEM; Hitachi S-4800, Japan). A cold field-emission scanning electron microscope (SEM; Regulus 8100, Japan) was used to analyse the morphology of core-spun yarns and the weaved fabric. The contact angles between absolute ethanol and the specimens were measured by a contact angle meter (DSA100, Krüss, Germany), wetting time was set to 60 s. Raman spectra were obtained using a laser Raman spectrometer (XploRA PLUS, Horiba, Japan) with a 638 nm activation laser as a radiation source. The rheological test of STF was conducted using an MCR 302 (Anton Paar, Austria) at 20 °C. Parallel plates with a diameter of 25 mm were used, and the gap between the plates was kept at 0.50 mm in the shear rate scanning range of 0.1–1000 s^−1^. Fourier-transform infrared spectra (FTIR; Bruker TERSOR37, Germany) were recorded from 4000 cm^−1^ to 400 cm^−1^ at a resolution of 4 cm^−1^. Thermogravimetric analysis (TGA; NETZSCH STA449F3, Germany) was conducted at 40–800 °C in a nitrogen atmosphere at a heating rate of 10 °C min^−1^. Differential scanning calorimetry (DSC; NETZSCH 200 F3, Germany) was performed at −45–60 °C in a highly purified nitrogen atmosphere at a heating rate of ±10 °C min^−1^. X-ray photoelectron spectroscopy (XPS) was conducted on a Genesis 60 spectrometer (Edax, USA) equipped with an Al-Kα radiation source (*hν* = 1486.4 eV). The crystallinity of the specimens was detected by X-ray diffraction (XRD; Rigaku D/MAX-gA, Japan) at room temperature in a 2*θ* range of 10°–60°.

### 2.6 Yarn pull-out test

**Fig. 2:**
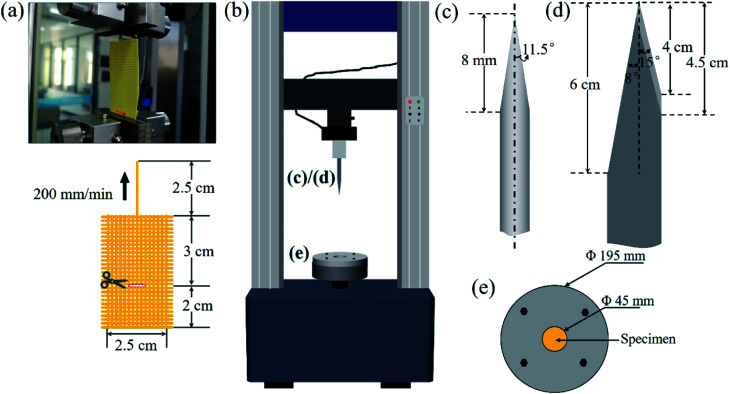
Schematic illustration of the yarn pull-out test: (a) yarn pull-out test setup and fabric dimension, (b) Hongda universal power machine, (c) spike impactor, (d) knife impactor, and (e) fabric holder.

The friction force between yarns is an important parameter that affects the impact resistance of fabrics. The friction force between yarns was estimated by pulling out yarns from the fabric. A universal testing machine (KY6800, KAIYAN, China) was used for the yarn pull-out test. When the pull-out rate was 3 mm s^−1^, the inter-yarn interface shear rate was about 600 s^−1^.^12^ The yarn pull-out rate was set at 200 mm min^−1^, exceeding the shear rate corresponding to the peak viscosity of STF/C_60_-FA. A single yarn was clamped by the upper clamp of the testing machine, and the bottom edge of the fabric specimen was fixed in the lower clamp (Fig. 2(a)). The upper clamp moved upward at a speed of 200 mm min^−1^ until the single yarn was completely pulled out. The dimensions of each part of the fabric specimen are indicated in Fig. 2(a). The yarn pull-out test of each specimen was measured 5 times and the median was selected as the final measurement result.

### 2.7 Stab resistance test

The stab resistance test of the core-spun yarn fabric was performed on a Hongda universal testing machine (Fig. 2(b)). The specific sizes of the impactors (spike and knife) are presented in Fig. 2(c) and (d). The specimen size was 10 cm × 10 cm, and each specimen was fixed on the base using four screws (Fig. 2(e)). The stab resistance test was performed according to the ASTM F1342-05 standard, and the puncture speed was set to 508 mm min^−1^. Each specimen was tested at least five times, and the average value was calculated after removing the extreme value. To keep the specimen thickness and areal density as similar as possible, five layers of AF, two layers of AF/PK, and two layers of AF/PK/STF/C_60_-FA were used. The specimen data are listed in Table 1.

**Table 1:** Specimen parameters for the punching experiment

Specimen	Thickness (mm)	Areal density (g m^−2^)	Layers
AF	0.30 ± 0.05	210 ± 10	5
AF/PK	0.75 ± 0.05	490 ± 10	2
AF/PK/STF/C_60_-FA	0.75 ± 0.05	505 ± 10	2

## 3. Results and discussion

### 3.1 Hydroxylated C_60_

**Fig. 3:**
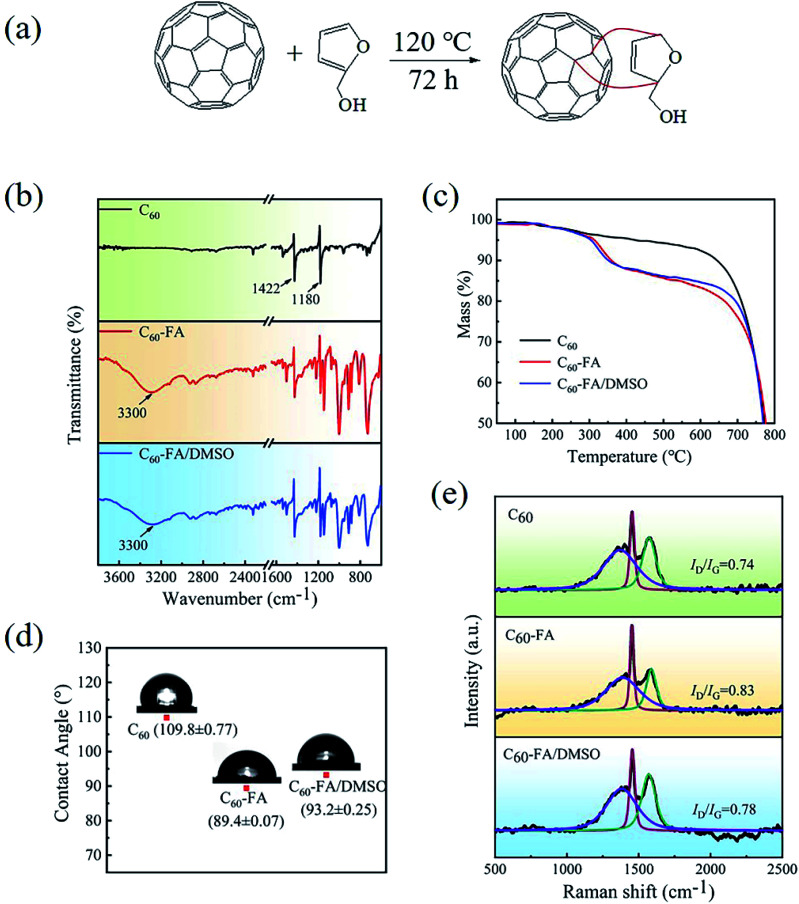
(a) Illustration of C_60_ functionalization by the D–A reaction, (b) FTIR spectra of C_60_, C_60_-FA, and C_60_-FA/DMSO, (c) TGA curves of C_60_, C_60_-FA, and C_60_-FA/DMSO, (d) contact angle data of C_60_, C_60_-FA, and C_60_-FA/DMSO, and (e) Raman spectra of C_60_, C_60_-FA, and C_60_-FA/DMSO.

Fig. 3(a) presents the D–A reaction between C_60_ and FA. C_60_ acted as a dienophile with more double bonds on the [6, 6] position,^37–39^ and FA acted as a diene. FA existed in a liquid state at room temperature. Fig. 3(b) displays the FTIR spectra of C_60_, C_60_-FA, and C_60_-FA/DMSO. The characteristic peaks of C_60_ appeared at 1422 cm^−1^ and 1180 cm^−1^.^40,41^ The characteristic peak of –OH (3300 cm^−1^) was detected on the FTIR spectra of C_60_-FA and C_60_-FA/DMSO, indicating the existence of the –OH functional group of FA in the C_60_ samples. The FA amounts of C_60_-FA and C_60_-FA/DMSO were measured by TGA (Fig. 3(c)). The decomposition onset temperature of C_60_ in the nitrogen atmosphere was about 557 °C, therefore, the mass losses of C_60_-FA and C_60_-FA/DMSO at about 300 °C could be assigned to the mass loss of grafted FA. At 550 °C, the mass losses of C_60_-FA and C_60_-FA/DMSO were 8.8 wt% and 7.0 wt%, respectively. According to the molar mass formula, 1 mol of C_60_ was grafted in 0.71 mol of FA in C_60_-FA, whereas 1 mol of C_60_ was grafted in 0.56 mol of FA in C_60_-FA/DMSO.

The contact angles of C_60_-FA and C_60_-FA/DMSO with ethanol were measured as 89.4 ± 0.07° and 93.2 ± 0.25°, respectively, which were lower than that of C_60_ (109.8 ± 0.77°) (Fig. 3(d)), indicating that the solubility of grafted C_60_ in the –OH containing solution was improved. Fig. 3(e) presents the Raman spectra of C_60_, C_60_-FA, and C_60_-FA/DMSO. The two prominent peaks at 1380 cm^−1^ and 1575 cm^−1^ could be attributed to the D band (vibration of sp^3^-hybridized carbon atoms) and the G band (in-plane vibration of sp^2^ carbon atoms), respectively.^33^ However, after the functionalization of FA, C_60_ produced more sp^3^ carbon atoms; thereby, the D band strength of C_60_ was improved. The *I*_D_/*I*_G_ ratios of C_60_-FA and C_60_-FA/DMSO were about 0.83 and 0.78, respectively, which were larger than that of C_60_ (0.74). The increase in the *I*_D_/*I*_G_ ratio provided additional support for the successful functionalization of C_60_. The solvent had little influence on the D–A between C_60_ grafted FA. In the absence of the solvent, the grafting amount was relatively higher because of a greater chance of the contact reaction between C_60_ and FA. Hence, C_60_-FA is selected for the following analysis.

### 3.2 Coaxial fibres with STF as the core

**Fig. 4:**
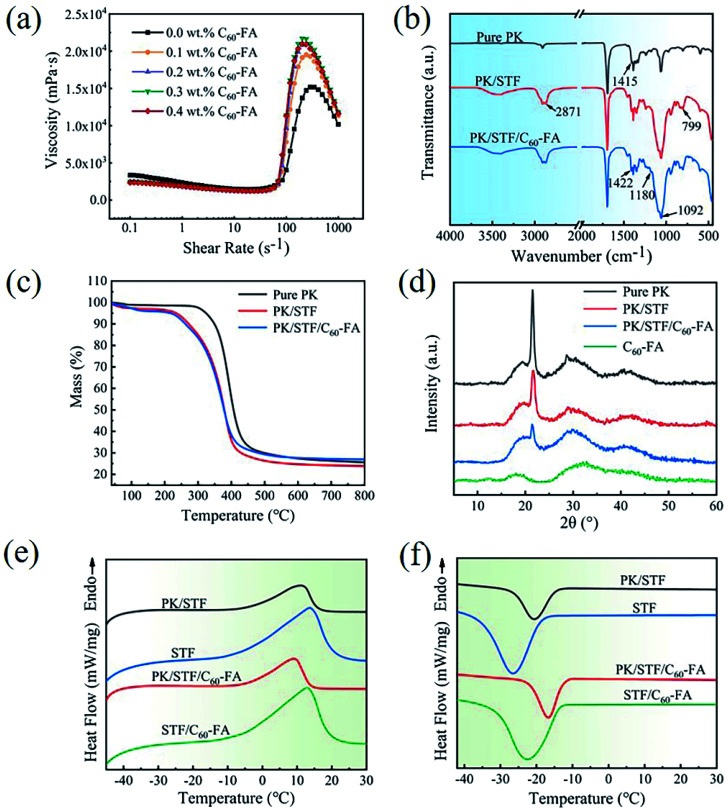
(a) Rheological curves of STF, (b) FTIR spectra of pure PK, PK/STF, and PK/STF/C_60_-FA fibres, (c) TGA curves of pure PK, STF/PK, and C_60_-STF/PK fibers, (d) XRD spectra of pure PK, PK/STF, PK/STF/C_60_-FA, and C_60_-FA, and (e) and (f) heating and cooling DSC curves of PK/STF, STF, PK/STF/C_60_-FA, and STF/C_60_-FA.

The rheological curves of the modified STF are displayed in Fig. 4(a). When 0.1 wt% of C_60_-FA was added, the thickening performance was significantly enhanced. When 0.3 wt% of C_60_-FA was added, the rheological performance was the strongest and the maximum viscosity increased by about 42.7%. The thickening effects of 0.4 wt% and 0.2 wt% of C_60_-FA were similar. It might happen because an excessive addition of C_60_-FA could cause particle agglomeration and reduce rheological properties. In particular, C_60_ has lubricity and could reduce the initial viscosity of the STF, which has never been seen in previous modifications. The specific rheological properties of the STF are summarized in Table 2. Table 3 compares the thickening ratio of the modified STF with other types of STF, and C_60_ manifested obvious advantages.

**Table 2:** Rheological properties of STF

Specimen	STF	0.1 wt% C_60_-FA	0.2 wt% C_60_-FA	0.3 wt% C_60_-FA	0.4 wt% C_60_-FA
Initial viscosity (Pa s)	3.36	2.35	2.37	2.46	2.48
Critical shear rate (s^−1^)	119.0	83.8	83.8	83.8	83.8
Critical viscosity (Pa s)	1.46	1.22	1.25	1.27	1.24
Peak shear rate (s^−1^)	289	242	242	203	203
Maximum viscosity (Pa s)	15.2	19.5	20.9	21.7	21.0
Thickening ratio	10.4	16.0	16.8	17.1	16.9
Peak viscosity increment (%)	—	28.3	37.5	42.7	38.2

**Table 3:** Comparison of the thickening ratios of different types of STF^a^

STF	Thickening ratio	References
1 wt% GO + 1 wt% CNTs	11.8	Lulu Liu *et al.* (2020)^31^
0.8 wt% MWCNTs	15.7	Minghai Wei *et al.* (2020)^27^
0.2 wt% MWCNTs	8.2	TingTing Li *et al.* (2019)^42^
0.5 wt% GO	<10	Mehdi Zojaji *et al.* (2021)^43^
0.01 wt% fMWNCTs:rGO = 1	<10	Hyun Taek Jeong *et al.* (2018)^29^
0.8 wt% MWCNTs	9.18	Mahdi Hasanzadeh *et al.* (2016)^44^
0.3 wt% GO	<10	Wenchao Huang *et al.* (2015)^45^
0.3 wt% C_60_-FA	17.1	This work

a Note: fMWNCTs: one-dimensional (1D) functionalized MWCNTs, rGO: two-dimensional (2D) reduced GO.

Fig. 4(b) exhibits the FTIR spectra of pure PK, PK/STF, and PK/STF/C_60_-FA fibres. In PK/STF composite fibres, the symmetrical stretching vibration of C–H in CH_2_ bonds was observed at 2871 cm^−1^, which was the characteristic peak of PEG molecular chains. Moreover, the characteristic peaks of SiO_2_ at 1092 cm^−1^ and 799 cm^−1^ confirm that the fibres contained STF. In contrast, the characteristic peaks of C_60_-FA appeared at 1180 cm^−1^ and 1422 cm^−1^, confirming that PK/STF/C_60_-FA composite fibres contained C_60_-FA.

The TGA curves of pure PK, STF/PK, and C_60_-STF/PK fibres are presented in Fig. 4(c). The composite fibres experienced two mass loss steps. The initial decomposition temperature of the first mass loss was about 258 °C, which was the decomposition temperature of PEG in STF. The initial decomposition temperature of the second mass loss was about 348 °C, which was close to the initial decomposition temperature of pure PK fibres. When the temperature reached 600 °C, the residual amounts of PK/STF and PK/STF/C_60_-FA composite fibres were 24.71% and 27.63%, respectively, and this difference could be attributed to the presence of C_60_-FA in PK/STF/C_60_-FA composite fibres.

The XRD spectra of the samples are displayed in Fig. 4(d). The characteristic peaks of PK at 21.66° and 29.19° appeared from the (110) and (210) crystal planes, respectively, at room temperature. The increase in the amorphous region area of STF/PK fibres at 21.66° could be ascribed to the superposition of the amorphous bun peak of SiO_2_ at about 22°. The diffraction peak of smaller C_60_-FA crystal grains at 32.2° was broad and had a low intensity. The amorphous peak area of PK/STF/C_60_-FA also increased, confirming that PK/STF/C_60_-FA contained C_60_-FA.

The DSC curves of pure PK, PK/STF, and PK/STF/C_60_-FA fibres are displayed in Fig. 4(e) and (f), and the obtained DSC data are summarized in Table 4. PEG is a common solid–liquid phase change material. Owing to the strong interaction between –OH groups on the surface of SiO_2_ and PEG molecules thus restrains the phase transition of PEG.^46,47^ The enthalpy change of PEG400 in STF was analysed to calculate the STF content in composite fibres (eqn (2)), and the corresponding results are presented in the last column of Table 4.2
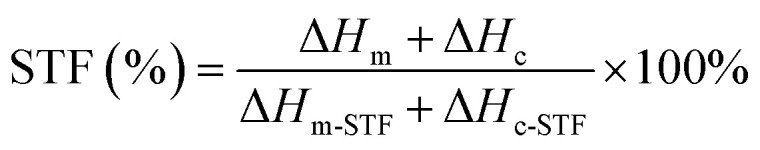
where, Δ*H*_m_ and Δ*H*_c_ are the melting enthalpy and crystallization enthalpy of composite fibres, respectively, Δ*H*_m-STF_ and Δ*H*_c-STF_ are the melting enthalpy and crystallization enthalpy of STF, respectively.

**Table 4:** Crystallization parameters of PK/STF, STF, PK/STF/C_60_-FA, and STF/C_60_-FA

Specimen	Δ*H*_m_ (J g^−1^)	Δ*H*_c_ (J g^−1^)	STF loading (%)
PK/STF	42.8	36.1	47.8
STF	82.3	82.7	—
PK/STF/C_60_-FA	35.7	34.3	42.5
STF/C_60_-FA	83.2	81.4	—

**Fig. 5:**
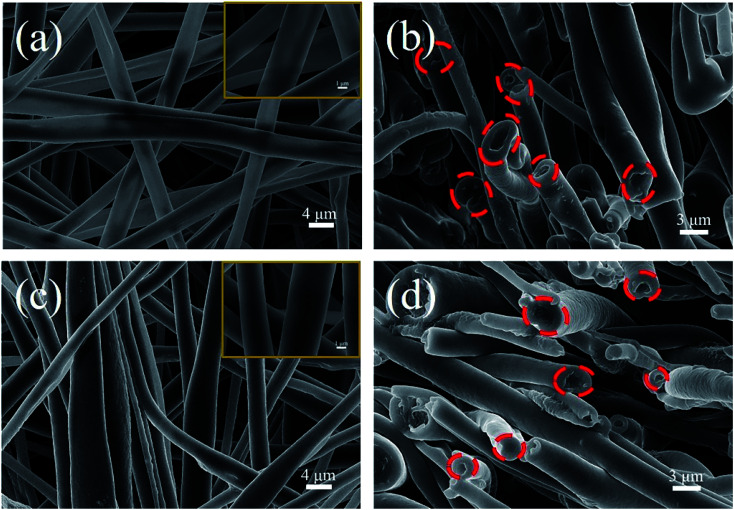
SEM images of the surface and cross-section of coaxial fibres: (a) and (b) PK/STF and (c) and (d) PK/STF/C_60_-FA.

The SEM images of the surface and cross-section of coaxial composite fibres are exhibited in Fig. 5. The fibre diameter was randomly measured 100 times using Nano Measurer software (Fig. 5(a) and (c)), and the diameter distribution statistics are presented in ESI.† The average diameters of PK/STF and PK/STF/C_60_-FA fibres were 3.29 μm and 3.22 μm, respectively, which were larger than that of pure PK fibres (1.88 μm). After the addition of C_60_-FA, the uniformity of fibre diameter distribution decreased due to the conductivity of C_60_. Fig. 5(b) and (d) present the cross-sectional view of sheath/core composite fibres (marked by red dashed lines). The central area of the cross-section of these composite fibres was selected for EDS analysis, and the corresponding data are listed in ESI.† The mass fractions of carbon in PK/STF and PK/STF/C_60_-FA composite fibres were 49.3 and 59.0, respectively (difference of ∼10%), indicating the existence of C_60_ in composite fibres.

### 3.3 Core-spun yarn

**Fig. 6:**
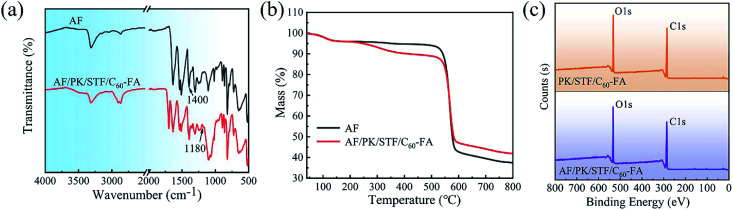
(a) FTIR spectra of AF and AF/PK/STF/C_60_-FA, (b) TGA curves of AF and AF/PK/STF/C_60_-FA, and (c) full-scale XPS spectra of PK/STF/C_60_-FA and AF/PK/STF/C_60_-FA.

Fig. 6(a) presents the FTIR spectra of AF and AF/PK/STF/C_60_-FA. The characteristic peaks of PK, STF, and C_60_-FA were detected in the FTIR curve of AF/PK/STF/C_60_-FA. The characteristic peak of C_60_-FA at 1422 cm^−1^ was superimposed with that of AF at 1400 cm^−1^, thus, it was not obvious. The TGA curves of AF and AF/PK/STF/C_60_-FA are exhibited in Fig. 6(b). In comparison to AF, AF/PK/STF/C_60_-FA had a significant mass loss around 260 °C, and it happened due to the decomposition of STF on the AF outer layer. The residual mass of AF/PK/STF/C_60_-FA was higher than that of AF because the decomposition temperature of SiO_2_ in STF was above 800 °C. No obvious Si 2s (154 eV) and Si 2p (103 eV) peaks were observed in the full-scale XPS spectra of PK/STF/C_60_-FA and AF/PK/STF/C_60_-FA (Fig. 6(c)). The silicon contents on the surface of PK/STF/C_60_-FA and AF/PK/STF/C_60_-FA were 0.82% and 1.36%, respectively, confirming that STF/C_60_-FA was stably wrapped on the fibre core without a leakage. The surface element contents of the specimens were re-tested after nearly 10 months, and almost no change was detected, indicating the excellent stability of the samples.

**Fig. 7:**
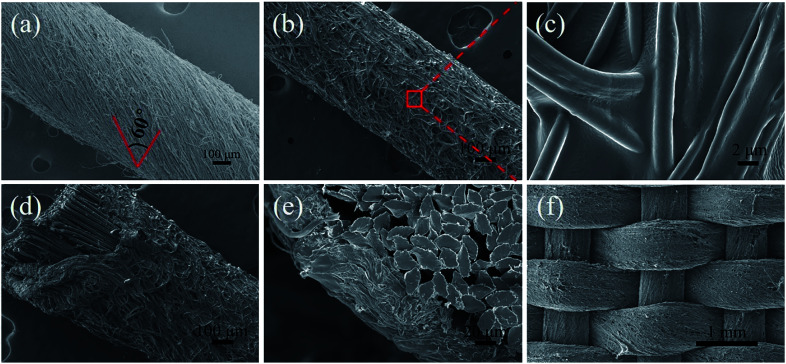
Core-spun yarn morphology of AF/PK/STF/C_60_-FA: (a) original core-spun yarns, (b) core-spun yarn after the hydrosol treatment, (c) surface fibres of core-spun yarn after the hydrosol treatment, (d) oblique cross-section of core-spun yarn, (e) cross-section of core-spun yarn, and (f) core-spun yarn fabric.

Fig. 7(a) displays the appearance of AF/PK/STF/C_60_-FA. During the experiment, the twisting degree of core-spun yarn increased with the reduced of the funnel rotation speed. When the speed was 300 rpm, coaxial fibres were perfectly coated on the core yarn surface with a good orientation and a uniform twist distribution. The twist was approximately 60°. Coaxial fibres on the core-spun yarn surface were fixed by hydrosol (Fig. 7(b)) to avoid abrasion in the weaving process. A closer inspection of the yarn surface (Fig. 7(c)) revealed that coaxial fibres significantly increased the roughness of the yarn surface, increasing the friction between yarns. Fig. 7(d) exhibits the oblique sectional view of core-spun yarns. The whole yarn section was nearly round. The thickness of the outer fibres was about 60 μm (Fig. 7(e)). The comparison presented that the amount of STF on the surface of the core-spun yarn was much higher than the amount of fibre adhesion on the surface of AF by impregnation method.^7,11,48^ The woven core-spun yarn fabric is presented in Fig. 7(f). It is observable that each yarn in the fabric was uniformly and completely covered by composite fibres, indicating that the fabrication process of core-spun yarns was stable and the woven fabric achieved the expected structure. The magnification for the SEM images was presented in the ESI.† In addition, the morphology of AF/PK/STF/C_60_-FA is similar to that of AF/PK, and the relevant data was listed in ESI.†

### 3.4 Yarn pull-out test

**Fig. 8:**
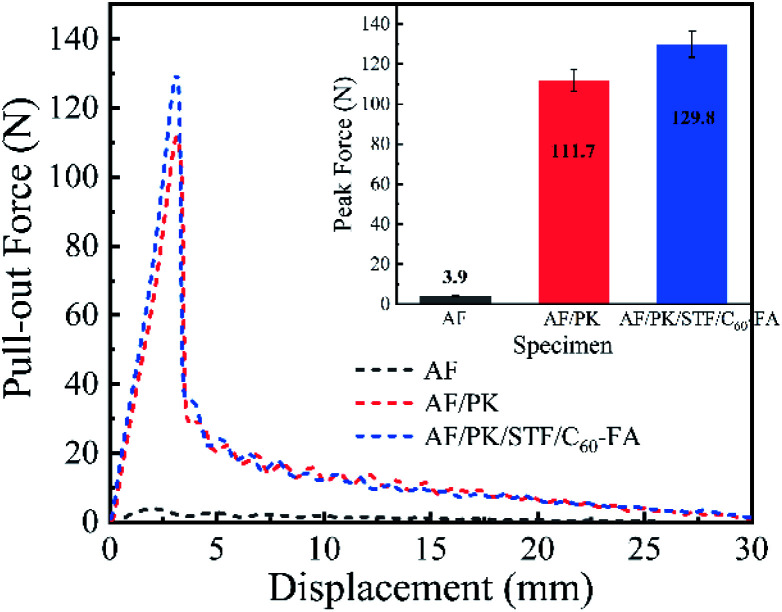
Pull-out force and displacement of AF, AF/PK, and AF/PK/STF/C_60_-FA.

The yarn pulling force and displacement of different fabrics are presented in Fig. 8. The yarn pull-out force of AF/PK was much higher than that of AF because PK fibres on AF increased the surface roughness of yarns. The yarn pull-out force of AF/PK/STF/C_60_-FA (129.8 N) was about 16.2% higher than that of AF/PK (111.7 N). It might happen because a shearing action occurred between adjacent yarns during the yarn pull-out process, thus, the STF of the coaxial fibre core underwent shear thickening, and the friction between yarns increased. Hence, AF/PK/STF/C_60_-FA is expected to have better puncture resistance.

### 3.5 Stab resistance test

**Fig. 9:**
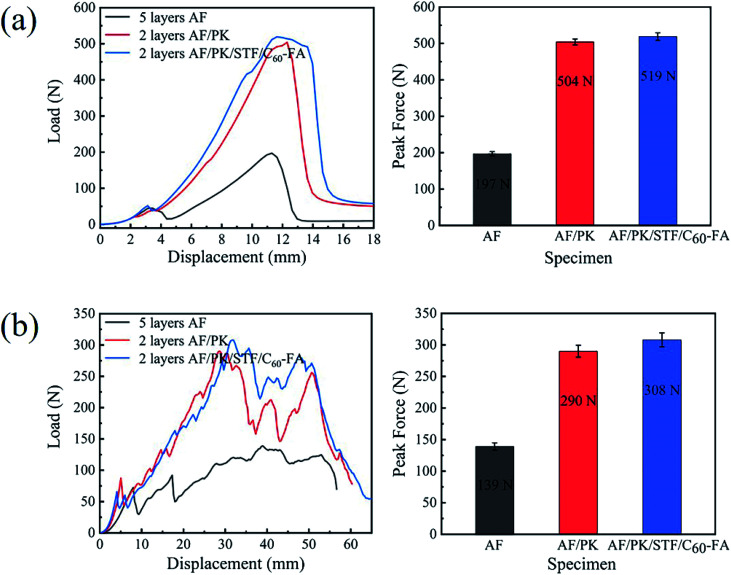
Load–displacement curves of AF, AF/PK, and AF/PK/STF/C_60_-FA: (a) spike stabbing and (b) knife stabbing.

Fig. 9(a) displays the load–displacement curves of AF, AF/PK, and AF/PK/STF/C_60_-FA during spike punching. The peak forces of AF, AF/PK, and AF/PK/STF/C_60_-FA were 197 N, 504 N, and 519 N, respectively. Under the similar thickness and surface density, the core-spun yarn fabric had better spike resistance. The peak force of AF/PK (504 N) was about 155.8% higher than that of AF (197 N), and the peak force of AF/PK/STF/C_60_-FA (519 N) was slightly higher than that of AF/PK. The integral area of the curve in Fig. 9(a) represents the energy absorbed by the fabric during the spike punching process.^49^ The higher the energy absorbed, the stronger the punching resistance of the fabric. The spike punching resistance of AF/PK was improved by about 196.7% as compared to that of AF, whereas the spike punching resistance of AF/PK/STF/C_60_-FA was enhanced by 29.4% as compared to that of AF/PK due to the effect of STF. The spike punching resistance of AF/PK/STF/C_60_-FA was improved by 283.9% as compared to that of AF.

Fig. 9(b) displays the load–displacement curves of AF, AF/PK, and AF/PK/STF/C_60_-FA during knife punching. The peak forces of AF, AF/PK, and AF/PK/STF/C_60_-FA were 139 N, 290 N, and 308 N, respectively. The peak force of AF/PK/STF/C_60_-FA was about 121.6% higher than that of AF. Fibre cutting was the main failure mode of the fabric during knife punching, which is different from the failure mode of fabric during spike punching. During the punching process, the tip of the knife first dug a small hole in the fabric and then the knife edge cut the fabric. The force on the fabric was mainly the shearing effect of the knife head on the fabric followed by the friction between the blade and the fabric. The integral area of the curve in Fig. 9(b) represents the energy absorbed by the fabric during the knife punching process. The knife punching resistance of AF/PK was about 103.3% higher than that of AF, whereas the knife punching resistance of AF/PK/STF/C_60_-FA increased by 13.2% as compared to that of AF/PK, owing to the effect of STF. The knife punching resistance of AF/PK/STF/C_60_-FA was improved by 130.1% as compared to that of AF/PK. The stab-resistance of the core-spun yarn fabric containing STF fabricated in the experiment was very obvious. The integration-related data are listed in ESI.†

It was found that the impactor shape greatly affected the punching resistance of the fabric by comparing the experimental results of cone puncture and knife puncture. The puncture resistance of the core-spun yarn fabric fabricated in this experiment is more advantageous to the cone impactor.

## 4. Conclusions

C_60_ was added to an STF for the first time to improve the thickening ratio. Composite fibres consisting of the STF were wrapped on the surface of aramid fibres to fabricate core-spun yarn fabric. The fabrication process of core-spun yarns was stable and continuous. The spike punching resistance and knife stabbing resistance of the core-spun yarn fabric were about 2.8 times and 1.3 times those of AF, respectively, under the similar fabric thickness and areal density. It had a breakthrough improvement effect on the impact resistance of the fabric. The experimental method in this paper solves the current problems of STF's moisture absorption, flow and adhesion on the fibre surface, and the content of STF in the fabric was more. Impact-resistant core-spun yarn fabric could provide a new direction for the development and research of soft armor in the future.

## Author contributions

Wenhua Cai: conceptualization, methodology, investigation, formal analysis, visualization, writing – original draft, writing – review & editing. Rong Zhang: methodology, supervision. Xuechen Wang: methodology writing – original draft. Xingxiang Zhang: conceptualization, investigation, formal analysis, writing – review & editing, supervision.

## Conflicts of interest

The authors declare no competing financial interest.

## Supplementary Material

RA-012-D2RA01095J-s001

RA-012-D2RA01095J-s002
