# Supplementary material for: Fabrication and characterization of impact-resistant core-spun yarn fabrics with a hydroxylated fullerene-strengthened shear thickening fluid
Source: RSC Adv. 2022 Apr 25;12(20):12507–16. doi: 10.1039/d2ra01095j (PMC9038179; doi:10.1039/d2ra01095j)
Supplement: RA-012-D2RA01095J-s002 [file RA-012-D2RA01095J-s002.pdf]

Electronic Supplementary Material (ESI) for RSC Advances.
This journal is © The Royal Society of Chemistry 2022

3.2 Coaxial fiber with a STF core

The diameter distribution of composite fibers is shown in Figure 1. The average diameters of PK/STF and PK/STF/C_{60}-FA were 3.29 μm and 3.22 μm, respectively, which was no obvious different.

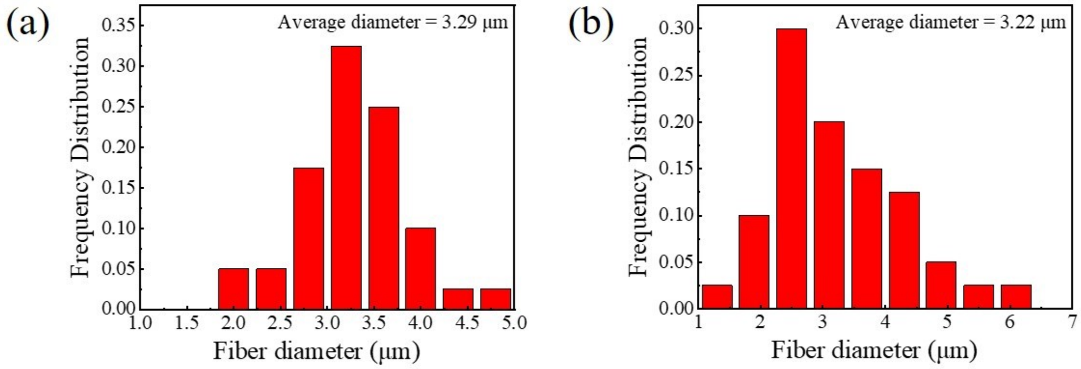

Figure 1. Diameter distribution of composite fibers. (a) PK /STF, (b) PK /STF/C_{60}-FA.

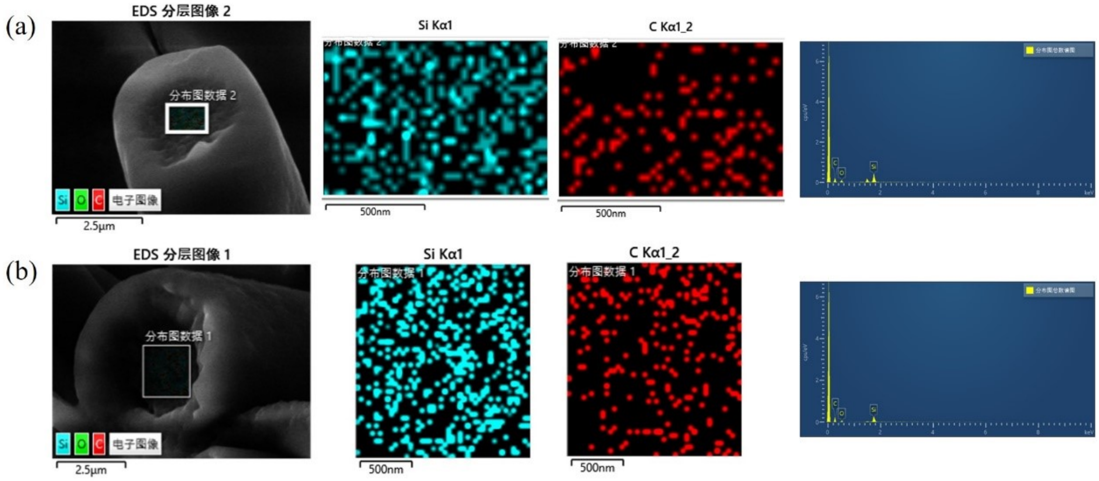

Figure 2. EDS analysis of composite fiber cross section. (a) PK /STF, (b) PK /STF/C_{60}-FA.

Select the central area of the composite fibers section where the sheath/core structure is not obvious for EDS energy spectrum analysis, as displayed in Figure 2. The mass fractions of carbon of PK/STF (Figure 2(a)) and PK/STF/C_{60}-FA (Figure 2(b)) composite fibers were 49.3 and 59.0, respectively, and the difference between the mass fractions of C was nearly 10%, indicating the existence of C_{60} in the composite fiber. It can also be explained from the side that the coaxial fiber has a high coaxial ratio.

3.3 Core-spun yarn

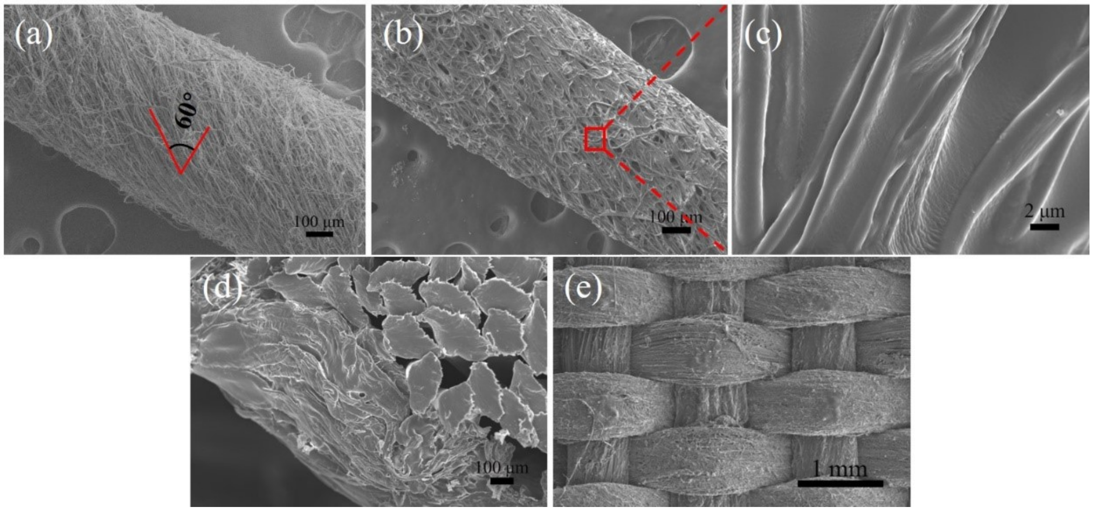

Figure 3. Core-spun yarn morphology of AF/PK: (a) Original core-spun yarn morphology, (b) Core-spun yarn after hydrosol treatment, (c) Surface fiber of core-spun yarn after treatment; (d) Cross section of core-spun yarn, (e) Core-spun yarn fabric.

The different parts of the core spun yarn and the appearance of the fabric are presented in Figure 3. The morphology of AF/PK is similar to that of AF/PK/STF/C_{60}-FA.

The magnification of SEM images: in Figure 5, (a) and (c) were magnified by 2000 times, (b) and (d) were magnified by 3000 times. In Figure 7, the magnifications of (a)-(f) are 100, 100, 5000, 100, 800, and 30 times in sequence.

3.5 Stab resistance test

The stab-resistance of the core-spun yarn fabric containing STF fabricated in the experiment was very obvious. The integration-related data are listed in Table 1.

Table 1 Integral data from stab resistance test.

Style	Specimen	Integration (J)
Spike stabbing	5 layers AF	0.9903
	2 layers AF/PK	2.9384
	2 layers AF/PK/STF/C_{60}-FA	3.8014
Knife stabbing	5 layers AF	4.8382
	2 layers AF/PK	9.8375
	2 layers AF/PK/STF/C_{60}-FA	11.1314
